# Anticancer Activity of Bee Venom Components against Breast Cancer

**DOI:** 10.3390/toxins14070460

**Published:** 2022-07-05

**Authors:** Na-Yoen Kwon, Soo-Hyun Sung, Hyun-Kyung Sung, Jang-Kyung Park

**Affiliations:** 1 Department of Obstetrics and Gynecology, College of Korean Medicine, Ga-Chon University, Seongnam-si 13120, Korea; kwonnay@gachon.ac.kr; 2 Department of Policy Development, National Institute of Korean Medicine Development, Seoul 04554, Korea; koyote10010@nikom.or.kr; 3 Department of Korean Medicine Pediatrics, School of Korean Medicine, Semyung University, Jecheon 27136, Korea; 4 Department of Korean Medicine Obstetrics and Gynecology, School of Korean Medicine, Pusan National University, Yangsan 50612, Korea

**Keywords:** bee venom, melittin, phospholipase A2, breast cancer

## Abstract

While the survival rate has increased due to treatments for breast cancer, the quality of life has decreased because of the side effects of chemotherapy. Various toxins are being developed as alternative breast cancer treatments, and bee venom is drawing attention as one of them. We analyzed the effect of bee venom and its components on breast cancer cells and reviewed the mechanism underlying the anticancer effects of bee venom. Data up to March 2022 were searched from PubMed, EMBASE, OASIS, KISS, and Science Direct online databases, and studies that met the inclusion criteria were reviewed. Among 612 studies, 11 were selected for this research. Diverse drugs were administered, including crude bee venom, melittin, phospholipase A2, and their complexes. All drugs reduced the number of breast cancer cells in proportion to the dose and time. The mechanisms of anticancer effects included cytotoxicity, apoptosis, cell targeting, gene expression regulation, and cell lysis. Summarily, bee venom and its components exert anticancer effects on human breast cancer cells. Depending on the mechanisms of anticancer effects, side effects are expected to be reduced by using various vehicles. Bee venom and its components have the potential to prevent and treat breast cancer in the future.

## 1. Introduction

Breast cancer is one of the most common cancers among women, accounting for 30% of all newly diagnosed cancers [1,2]. According to the American Cancer Society, approximately 2.3 million new patients with breast cancer were diagnosed and 685,000 deaths had resulted from breast cancer, making it the fifth leading cause of cancer mortality worldwide in 2020 [3]. Female breast cancer has a 5-year relative survival rate of 90% for all stages combined, which represents the third highest survival rate among major cancers in the United States [4]. However, as the stage progresses, the survival rate also rapidly decreases [5].

Breast cancer can be classified into three subtypes, depending on the presence of molecular markers: hormone receptor positive/human epidermal growth factor receptor-2 gene (ERBB2) negative, ERBB2 positive, and triple-negative [6]. These subtypes determine the recurrence rate and treatment strategies, including endocrine therapy, chemotherapy, surgery, radiation therapy, or a combination of these [6].

Standard endocrine therapy involves the intake of medications that competitively inhibit the binding of estrogen to its receptors or decrease the levels of circulating estrogen by inhibiting the conversion of androgens to estrogen. The side effects of these medications include hot flashes, uterine cancer, arthralgia, myalgia, and osteoporosis.

In several cases, chemotherapy is an essential treatment for preventing recurrence by disrupting mitosis or DNA replication. Patients undergoing this therapy complain of asthenia, edema, myalgia, and leukemia.

Depending on the metastasis of breast cancer cells, surgical treatment varies in terms of the degree of removal from the local region to the entire breast with the axillary lymph nodes [7,8]. Surgery can lead to lymphedema by interrupting the lymphatic drainage system or causing nerve injury.

Radiation therapy, particularly post-mastectomy radiation therapy, decreases the risk of local recurrence and improves the absolute survival benefit [9]. Nevertheless, in a decade-long study, loco-regional recurrence was observed and complaints of arm lymphedema were confirmed, including severe symptoms [10]. In order to reduce the side effects of these standard treatments, cancer patients seek complementary and alternative medicine.

Natural products from animals and plants have been applied as therapeutic agents to combat various diseases [11]. Toxins that have evolved to damage other living organisms have been clinically evaluated in the context of oncological diseases [12]. For instance, botulin toxin has an anesthetic effect in cancer radiotherapy and can not only suppress tumor growth but can also trigger apoptosis in cancer cells [13].

Bee venom contains many active components, including melittin, mast cell degranulating peptide, apamin, enzymes (e.g., phospholipase A2, hyaluronidase), and amino acids [14]. Melittin, the chief component of bee venom, accounts for 40–60% of bee venom composition and is the major substance that produces pain [15]. Melittin can be easily inserted into membranes by pore formation and perturbation in a non-selective manner, resulting in antimicrobial and antitumor activities and hemolysis [14]. Therefore, bee venom cannot be used without a proper delivery vehicle. To date, several studies on bee venom have been conducted to develop the right vehicle in order for the appropriate dose to reach cancer cells.

Bee venom and melittin have been confirmed to be effective in ovarian cancer, prostate cancer, and human malignant hepatocellular carcinoma [16,17,18]. Additionally, studies have shown the therapeutic effects of bee venom and melittin on breast cancer. However, as cell lines, vehicles, and outcomes vary, integrated research should be conducted.

In this review, we discussed the published in vitro studies on breast cancer treatment with bee venom and melittin and comprehensively identified the mechanisms underlying the treatment and prevention of breast cancer metastasis.

## 2. Results

**Figure 1:**
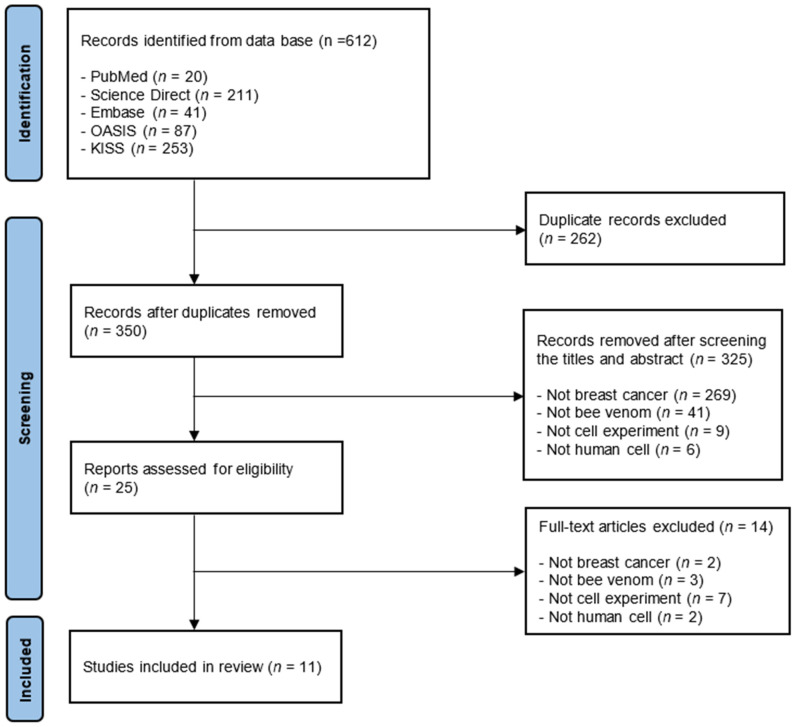
Flow Diagram of the Review.

The search resulted in the discovery of 612 studies. Out of these studies, 262 duplicates were excluded from the meta-analysis. The titles and abstracts were checked, and those studies that did not meet the inclusion criteria were excluded. Subsequently, only studies that fulfilled the selection criteria were selected by checking the entire paper in 25 studies. Finally, a total of 11 studies were analyzed (Figure 1).

### 2.1. Analysis of Experimental Methods

Bee venom was administered to breast cancer cells in six studies [19,20,21,22,23,24], whereas melittin or processed melittin was administered to breast cancer cells in seven studies [20,21,22,25,26,27,28]. Among them, three studies compared the results of melittin and bee venom administration [20,21,22]. There was only one study on phospholipase A2 from bee venom [29]. All studies, except one, attempted to confirm the results according to the dose [19,20,21,22,23,24,25,26,28,29]. On the other hand, three studies confirmed the results according to the duration of administration [22,23,26] (Table 1).

### 2.2. Analysis of Experimental Results

As bee venom and its components are known to cause toxic effects and apoptosis in cancer cells, most studies have confirmed the mechanisms related to these. Their experimental results confirmed that breast cancer cells were more effectively eliminated in the experimental group than in the control group. With respect to studies comparing bee venom and melittin, one study reported that the effect of melittin was greater than that of bee venom [20] and another study showed that the effect of bee venom was due to melittin [21]. A study targeting specific proteins in cancer cells reported that they showed higher selectivity [27] (Table 2).

## 3. Discussion

### 3.1. Cytotoxic Activity

As cancer cells are less likely to develop resistance to a membrane pore former, combining a chemotherapeutic medication with melittin could be an effective synergistic treatment [20].

Hematyar et al. [25] showed that all drug formulations, such as melittin, doxorubicin, and doxorubicin/melittin-loaded citric acid-functionalized Fe_3_O_4_ magnetic nanoparticles (doxorubicin/melittin-loaded CA-MNPs), decreased the cell growth in a dose-dependent manner and that doxorubicin and melittin delivered together exhibited a synergistic effect on MCF-7 breast cancer cell proliferation. Because anticancer drugs were more effectively delivered into cells via internalized nanoparticles at the same dose, doxorubicin/melittin-loaded CA-MNPs had better cytotoxic action than free doxorubicin/melittin (1:4).

Niosomes, which are non-ionic surfactant vesicles, have the ability to directly target tumor cells by increasing efficacy and lowering the side effects [30]. The negative effects of drug protection, high stability, and long shelf life are among the most prominent reasons for the delay in drug delivery to target cells in pharmacological research [31]. In order to prevent these side effects, Moghaddam et al. [26] used niosomes as nanocarriers for melittin to enhance the anticancer effects and prevent the hemolytic side effects. They proved that melittin-loaded nanoniosomes had higher anticancer effects and fewer side effects in breast cancer cell treatment.

Because melittin, a peptide found in bee venom, is known to cause nonspecific cytotoxicity and hemolysis, it is important to reduce the dosage of melittin for cancer treatment. Shaw et al. [28] attempted to lower the dosage of melittin by combining melittin with plasma-treated phosphate-buffered saline (PT-PBS), which can induce cancer cell death via oxidative stress-mediated pathways. Melittin alone exerted a dose-dependent cytotoxic effect, apoptosis, and lipid peroxidation in MCF-7 cells. However, when synthesized with PT-PBS, a synergistic effect was observed. As melittin is not oxidized by plasma, this effect is thought to be attributable to the improved potential of melittin through the cell membrane during plasma-induced oxidation of phospholipids.

Cell-based experiments are among the most important studies for confirming the efficacy and mechanism of drugs. Cell culture, which is the most critical part of cell-based experiments, is the basis for cell responses to drugs, compounds, etc. [32]. Several experiments are based on two-dimensional (2D) cell culture. However, because this provides only a uniform environment, the need for three-dimensional (3D) cell culture that can mimic the microenvironment of normal and cancer cells has been raised. A 3D cell culture is different from a 2D cell culture with respect to morphology, proliferation, and stage of cell cycle, and cancer studies using the 3D culture have already been conducted [33,34].

Kamran et al. [19] administered bee venom to MCF-7 cells in proportion to the dose in order to confirm the cytotoxic and apoptotic effects of bee venom. The results regarding the reduction of cell viability and the inhibition of cell growth were confirmed in a 3D culture. Similar to other studies, higher resistance to the cytotoxic effect of bee venom was observed in a 3D culture than in a 2D culture.

### 3.2. Apoptosis Activity

Apoptosis is a complex human defense mechanism that occurs under genetic control due to specific stages of occurrence, DNA damage, and viral infection [35]. It plays an important role in removing damaged cells at an individual conservation level and can be the main cause of deviation from the normal cell cycle [36].

Yeo et al. [24] explored the apoptotic effect of bee venom in MCF-7 cells by determining the coefficient of the number of living cells, morphological changes, biochemical changes, and gene expression changes in MCF-7 cells. Taken together, their results indicated that the suppression of human breast cancer cell proliferation caused by bee venom was linked to the induction of apoptosis. Jung et al. [23] attempted to demonstrate the effects of bee venom treatment by conducting a multivariate analysis. Bee venom had an effect on MDA-MB-231 cells in a concentration- and time-dependent manner through cell death-related processes involving protein denaturation and degradation, as well as DNA fragmentation.

Similarly, melittin is amphipathic and capable of disrupting the integrity of the tumor cell membrane bilayer, leading to flaws, disruption, or pore formation. Despite the exceptional anticancer effect of melittin, it is known to be toxic to normal cells, and an appropriate vehicle is required to produce the therapeutic effect. Nevertheless, Sharkawi et al. [20] showed that melittin could be toxic to tumor cells and that the dose worked just before it affected the normal cells. Furthermore, as confirmed by other studies, Sharkawi et al. [20] reported that bee venom and melittin caused cancer cell apoptosis by adjusting the genes related to apoptosis such as p53, Bax, and Bcl-2.

### 3.3. Cell Targeting

A previous study confirmed a significantly increased gene expression of fibroblast activation protein-α (FAP) compared with normal cells [37]. LeBeau et al. [27] evaluated FAP, a tumor stromal antigen overexpressed by cancer-associated fibroblasts, as a tumor-specific target [38]. Their study revealed that despite the function of FAP in tumors, the enzyme activity of FAP could be used to selectively activate high-intensity cytotoxins in peritumoral injection. This activation could lead to the death of tumor cells and produce a synergistic effect that causes tumor death within and around the stromal compartment.

While the effectiveness of cell targeting has been confirmed, it has a limitation in that cell targeting should be administered intratumorally and within the organ. Further studies are required to confirm its effectiveness according to the administration method.

### 3.4. Regulating Gene Expression

Matrix metalloproteinases (MMPs) are a group of enzymes required for extracellular matrix decomposition for cancer cell growth at metastatic sites [39]. MMP-9 plays a key role in the invasion and spread of human cancer cells [40].

Cho et al. [21] reported that bee venom did not abolish the expression of tissue inhibitors of metalloproteinases-1 and -2 and directly inhibited the ability of MCF-7 cells to invade and move by suppressing the expression of MMP-9. The inhibition of MMP-9 enzyme activity was caused by the inhibition of p39, JNK, and NF-Kb expression; among the components of the bee venom, melittin caused this effect.

Triple-negative breast cancer and human epidermal growth factor receptor-2 (HER2)-positive breast cancer are the most common breast cancers. Anti-HER2 treatment increases the survival rate of patients with early HER2-positive cancer. However, when it has progressed to the end of the stage, it becomes resistant to drugs and is therefore difficult to treat. Therefore, research on alternative methods for aggressive breast cancer treatment is required [41,42].

Duffy et al. [22] showed that bee venom and melittin dynamically regulated the downstream signaling pathway of breast cancer cells by inhibiting the phosphorylation of ligands of the epidermal growth factor receptor (EGFR) and HER2. Furthermore, melittin reacted more specifically to HER2- and EGFR-overexpressing breast cancer cells and showed greater toxicity than bee venom.

### 3.5. Cell Lysis

Monocyte-derived dendritic cells (moDCs), which are produced in peripheral blood precursor cells filled with tumor lysates or antigen, induce antitumor immune reactions when they are re-injected into patients [43]. In a previous study, it was confirmed that phospholipase A2 causes the maturation of moDCs through enzyme activation and NF-kB, activating protein-1, a nuclear factor of activated T-cells [44]. Putz et al. [29] attempted to determine the synergistic effect between phospholipase A2 (bv-sPLA2) and phosphatidylinositol-(3,4)-bisphosphate (PtdIns (3,4) P2) occurring during maturation of immunostimulatory moDCs mediating tumor cell lysis.

To quantify the amount of cell lysis, data were obtained by measuring [^3^H] thymidine incorporation. Although the incorporation of [^3^H] thymidine does not directly measure lytic capacity, it is a sensitive approach for detecting the proliferation of small numbers of unlysed cells that survive combined treatment. Putz et al. [29] identified T-47D cell inhibition and synergistic effects of bv-sPLA2 and PtdIns(3,4)P2, suggesting the possibility of an antitumor vaccine.

## 4. Conclusions

Breast cancer represents the most common malignancy among women worldwide, and the number of women diagnosed with breast cancer is increasing yearly due to the development of diagnostic devices and changes in lifestyle. Surgery and anticancer therapy are performed as general breast cancer treatments; nonetheless, the quality of life of patients during treatment decreases because of the side effects.

Various treatment methods are being studied to reduce the capacity of these treatments and different toxins are being investigated for their potential as anticancer agents. The bee venom contained in a honeybee’s solitary bag is a substance composed of approximately 40 active ingredients and has been used to treat related diseases because of its analgesic and anti-inflammatory properties.

Recently, the possibility of treatment has expanded to chemotherapy, and research on prostate, ovarian, and breast cancers is being actively conducted. In the case of ovarian and prostate cancers, a review article revealing the mechanism underlying the anticancer effects of bee venom and its components has been published. However, a review article focusing on breast cancer has not yet been published. Accordingly, the present study attempted to collect and analyze published experimental studies on human breast cancer to identify the effects of bee venom and its components on breast cancer cells and to confirm the underlying mechanism.

In this study, we confirmed that bee venom controls the metastasis of breast cancer cells and lowers cell viability in proportion to the dose and time. Furthermore, we identified cytotoxicity, apoptosis, targeting, gene expression regulation, and cell lysis as the mechanisms of breast cancer cell inhibition. The hemolytic effect, which is the most worrisome side effect of bee venom, can be mitigated by increasing selectivity, adjusting the dose to an appropriate amount, or utilizing the preventive effect of moDCs.

## 5. Materials and Methods

### 5.1. Data Sources and Searches

In March 2022, a study on breast cancer and bee venom treatment was conducted using the following electronic databases: MEDLINE (PubMed), Science Direct, Excerpta Medica Database (EMBASE), Korean Studies Information Service System (KISS), and Oriental Medicine Advanced Searching Integrated System (OASIS). We used both MeSH terms and free text words. A combination of keywords included bee venom (“bee venom”/exp OR “bee venom” OR “melittin”/exp OR “melittin”) and breast cancer (“breast cancer”/exp OR “breast cancer” OR “breast carcinoma”/exp OR “breast carcinoma” OR “Breast Neoplasms” OR “BRCA2 Protein” OR “BRCA1 Proteins”) and a combination of them. There were no restrictions in publication time.

### 5.2. Study Selection

We included experimental studies that evaluated the anti-cancer effect of bee venom on human breast cancer cells. We excluded clinical studies (randomized controlled trials, case studies, case series, or case-controlled trials), animal studies, surveys, and reviews. There were no restrictions in bee venom interventions.

### 5.3. Data Extraction

Three authors independently extracted data using pre-defined inclusion criteria. Further, two independent reviewers collected data regarding first author, anticancer agent, cancer cell, dose, duration of experiment, mechanism, method, and main results. In case of insufficient outcome data, the corresponding authors were contacted whenever possible. Disagreements were resolved.

## Figures and Tables

**Table 1:** Experimental Methods of Studies.

First Author (Publication Year)	Anticancer Agent	Cancer Cell	Dose	Duration of Experiment
Kamran et al., (2019) [19]	BV	MCF-7	2.5, 5.0, 7.5, 10, 12.5 (μg/mL)	24 h
Sharkawi et al., (2015) [20]	BV MEL Combination of L-amino acid oxidase from snake venom and MEL	MCF-7	20, 100 (μg/mL)	24 h
Hematyar et al., (2018) [25]	MEL and doxorubicin loaded onto citric acid-functionalized Fe_3_O_4_ magnetic nanoparticles	MCF-7	0.01–250 (μg/mL^−1^)	48 h
Moghaddam et al., (2021) [26]	MEL MLN Empty niosome	SK-BR-3	8, 16, 32, 64, 128, 256 (μg/mL)	48, 72 h
LeBeau et al., (2009) [27]	Modified promelittin	MCF-7	-	72 h
Cho et al., (2010) [21]	BV MEL	PMA-induced MCF-7	0.5, 1, 2, 3, 4, 5 (μg/mL)	24 h
Putz et al., (2006) [29]	Phospholipase A2 from bv Phosphatidylinositol-(3,4)-bisphosphate	T47D	10 (μg/mL)/10 (μM)	32 h
Duffy et al., (2020) [22]	BV MEL	TNBC (SUM159, SUM149) HER2-enriched breast cancer cell lines (MDA-MB-453, SK-BR-3) Luminal breast cancer cells (MCF7, T47D)	BV: 4, 5, 6, 7 μg/mL MEL: 2, 3, 4, 5 μg/mL	Caspase-3: 18, 24 h Flow cytometry analysis, cell viability, live-cell confocal microscopy, scanning electron microscopy: 1 h
Jung et al., (2018) [23]	BV	MDA-MB-231	Cytotoxicity: 2.5, 5.0, 7.5, 10, 12.5, 15 μg/mL Apoptotic cell death, Raman spectroscopic analysis, morphological changes: 0.7, 1.5, 3 μg/mL	Cytotoxicity: 12, 24, 48, 72 h Apoptotic cell death, Raman spectroscopic analysis, morphological changes: 12, 24, 48 h
Shaw et al., (2019) [28]	MEL PT-PBS Combination of PT-PBS and MEL	MCF-7	0.6, 1.2, 2.5, 5, 10 μg/mL	24 h
Yeo et al., (2003) [24]	BV	MCF-7	0.001, 0.01, 0.1, 1 μg/mL	24 h

BV—bee venom; MEL—melittin; MLN—melittin-loaded niosome; PMA—phorbol-12-myristate-13-acetate; PT-PBS—plasma-treated phosphate-buffered saline; TNBC—triple-negative breast cancer.

**Table 2:** Analysis of Experimental Results.

First Author (Publication Year)	Mechanism	Method	Main Results
Kamran et al. [19]	Cytotoxic and apoptotic effects	Cell viability Neutral red uptake Reactive nitrogen intermediates Reduced glutathione Catalase enzyme activity Alkaline comet assay Caspase-3 activity	CBV (in dose-dependent manner) NO production↑, caspase-3activation↑ MCF-7 viability↓, catalase activity↓, glutathione content↓ In assessing DNA damage, the cytotoxicity of CBV in MCF-7 cells was shown in a dose-dependent manner
Sharkawi et al. [20]	Cytotoxic and apoptotic effects	Cytotoxicity assays Apoptotic evaluation Cell cycle analysis	Cytotoxic activity of BV: MCF-7 cells > Normal cells Cytotoxic activity in MCF-7 cells: MEL > CBV MEL: Expression of p53↑, Bcl-2↑ BV: Expression of p53↑, Bcl-2↑, Bax↑ MEL increased the activity of phospholipase A2 from snake venom, exhibiting cooperative activity on the expression of p53 and Bax in MCF-7 cells
Hematyar et al. [25]	Cytotoxic effect	Cytotoxicity assays	Cell growth was reduced by all drug formulations in a concentration-dependent manner Cytotoxicity: DOX/MEL-loaded CA-MNPs > free DOX/MEL (1:4) > free DOX, free MEL
Moghaddam et al. [26]	Cytotoxic and apoptotic effects Prevention of cell migration required for cancer cell proliferation and metastasis	Cell proliferation Wound healing assay Soft agar colony assay Flow cytometry analysis Real-time PCR for gene expression	Inhibitory impact of SK-BR-3 (in dose- and time-dependent manner): niosomal formulation > free drug solution Cell migration of SK-BR-3: MEL > MLN Scratch width of SK-BR-3: Empty noisome, MEL, MLN > Control/MLN > MEL Decrease of colony number of BC cells: Empty noisome, MEL, MLN > Control/MLN > MEL Percentage of apoptosis: MLN > MEL > Control Expression of caspase-3, caspase-9, Bax: MLN, MEL, Empty noisome > Control/MLN > MEL Expression of bcl-2, MMP-2, MMP-9: MLN, MEL, Empty noisome < Control/MLN < MEL
LeBeau et al. [27]	Targeting FAP	FAP promelittin protoxins destroy human breast cancer cell lines that express FAP	Toxicity of MEL: No selectivity, FAP(−)↑, FAP(+)↑ Toxicity of modified promelittin: FAP(−)↓, FAP(+)↑ Ac-FAP6, FAP2 with DPP4 resistance by adding an NH2-terminal glycine acetylated to the FAP2 peptide, had the highest selectivity and efficiency
Cho et al. [21]	Regulation of MMP-9 expression during breast cancer cell invasion and metastasis	Cytotoxicity Matrigel invasion Wound healing assay Zymography Western blot analysis RT-PCR	MEL in BV ingredient suppressed cell invasion and migration in a dose-dependent manner by inhibiting PMA-induced MMP-9 gene activation via pathways such as JNK, p38, MAPK, and NF-KB
Putz et al. [29]	Massive cell lysis that reduces the number of cells with proliferative capacity	Inhibition of [^3^H] thymidine incorporation	Single treatment with PtdIns (3,4) P2 or bv-sPLA2 was effective in T-47D cells by inhibiting their proliferation Bv-sPLA2 and PtdIns (3,4) P2 had a comparable synergistic effect of inhibiting T-47D by affecting [^3^H] thymidine incorporation
Duffy et al. [22]	Induction of cell death and suppression of EGFR and HER2 activation by interfering with the phosphorylation of these receptors in the plasma membrane of breast cancer cells	Anticancer efficacy and selectivity	BV, MEL diminished the viability of BC cells and eliminated BC cells in a dose-dependent manner by enhancing the specificity for aggressive murine tumor cell lines RGD enhanced the breast cancer targeting of melittin BV and MEL impaired the RTK-associated signaling pathways by preventing the ligand-dependent activation of EGFR and HER2 in BC cells on SK-BR-3, SUM159
Jung et al. [23]	Apoptosis	Cytotoxicity Apoptotic cell death Morphological changes Raman spectra	BV: Proliferation of MDA-MB-231 cells↓, protein levels of caspase-8↓, caspase-9↓, caspase-3↓, morphological deformation↑, averaged Raman spectra↑in MDA-MB-231 cells in a time- and dose-dependent manner
Shaw et al. [28]	Combination of oxidative stress-medicated pathways and cytotoxicity	Cell viability Cell death Flow cytometry analysis Lipid peroxidation by MDA assay and fluorescent probe Mass spectrometry analysis	MEL: Cytotoxic effect↑, apoptosis/necrosis↑, lipid peroxidation↑ in MCF-7 cells Combination of MEL and PT-PBS: Synergistic effect of those effects and generated covalent alteration of proteins and nucleic acids inducing oxidative stress-mediated cell death There were no variations in MEL oxidation levels between control and plasma treatments, and there was no evidence of an increase in the number of oxidations with time
Yeo et al. [24]	Apoptosis	Cell growth and viability Morphological changes Induction of apoptosis and degradation of β-catenin in bee venom-treated MCF-7 cells Inhibition of Bcl-X_S/L_ and induction of Bax expression Levels of cell-cycle regulatory gene products and tumor suppressors	BV: MCF-7 cell viability↓(in a dose-dependent manner), β-catenin↓(in a dose-dependent manner), Bcl-X_S/L_↓, cyclin B1↓, cyclin C↓, morphological deformation↑(in a dose-dependent manner), BV cell death↑, Bax expression↑(in a dose-dependent manner), p53 expression↑, Cdk inhibitor p31↑

CBV—crude bee venom; NO—nitric oxide; BV—bee venom; MEL—melittin; DOX/MEL—doxorubicin/melittin; BC—breast cancer; CA-MNPs—citric acid-functionalized Fe_3_O_4_ magnetic nanoparticles; EGFR—epidermal growth factor receptor; FAP—fibroblast activation protein-α; MMP—matrix metalloproteinases; MLN—melittin-loaded noisome; RGD—tripeptide consisting of arginine, glycine, aspartate.

## Data Availability

Data sharing not applicable.

## References

[1] Berek J.S. (2019). Berek & Novak’s Gynecology.

[2] Ahmad A. (2019). Breast Cancer Metastasis and Drug Resistance.

[3] Sung H.A., Ferlay J., Siegel R.L., Laversanne M., Soerjomataram I., Jemal A., Bray F. (2021). Global cancer statistics 2020: GLOBOCAN estimates of incidence and mortality worldwide for 36 cancers in 185 countries. CA Cancer J. Clin..

[4] Siegel R.L., Miller K.D., Fuchs H.E., Jemal A. (2021). Cancer statistics 2021. CA Cancer J. Clin..

[5] The American Cancer Society Medical and Editorial Content Team (2021). Understanding a Breast Cancer Diagnosis.

[6] Waks A.G., Winer E.P. (2019). Breast cancer treatment: A review. JAMA.

[7] Ridner S.H. (2013). Pathophysiology of lymphedema. Semin. Oncol. Nurs..

[8] Ducic I., Zakaria H.M., Felder J.M., Fantus S. (2014). Nerve injuries in aesthetic breast surgery: Systematic review and treatment options. Aesthet. Surg. J..

[9] Lyons J.A., Sherertz T. (2014). Postmastectomy radiation therapy. Curr. Oncol. Rep..

[10] Mignot F., Quero L., Guillerm S., Benadon B., Labidi M., Cuvier C., Giacchetti S., Lorphelin H., Cahen-Doidy L., Teixeira L. (2022). Ten-year outcomes of hypofractionated postmastectomy radiation therapy of 26 Gy in 6 fractions. Int. J. Radiat. Oncol. Biol. Phys..

[11] Gajski G., Madunic J., Madunic I.V., Zovko T.C., Rak S., Breljak D., Osmak M., Vrhovac V.G. (2017). Anticancer effects of natural products from animal and plant origin. Biomed. Res. Ther..

[12] Shapira A., Benhar I. (2010). Toxin-based therapeutic approaches. Toxins.

[13] Grenda T., Grenda A., Krawczyk P., Kwiatek K. (2022). Botulinum toxin in cancer therapy-current perspectives and limitations. Appl. Microbiol. Biotechnol..

[14] Wehbe R., Frangieh J., Rima M., El Obeid D., Sabatier J.M., Fajloun Z. (2019). Bee venom: Overview of main compounds and bioactivities for therapeutic interests. Molecules.

[15] Chen J., Guan S.M., Sun W., Fu H. (2016). Melittin, the major pain-producing substance of bee venom. Neurosci. Bull..

[16] Moga M.A., Dimienescu O.G., Arvătescu C.A., Ifteni P., Pleş L. (2018). Anticancer activity of toxins from bee and snake venom-an overview on ovarian cancer. Molecules.

[17] Badawi J.K. (2021). Bee venom components as therapeutic tools against prostate cancer. Toxins.

[18] Li B., Gu W., Zhang C., Huang X.Q., Han K.Q., Ling C.Q. (2006). Growth arrest and apoptosis of the human hepatocellular carcinoma cell line BEL-7402 induced by melittin. Onkologie.

[19] Kamran M.R., Zargan J., Keshavarzalikhani H., Hajinoormohamadi A. (2020). The comparative cytotoxic effects of *Apis mellifera* crude venom on MCF-7 breast cancer cell line in 2D and 3D cell culture. Int. J. Pept. Res. Ther..

[20] Sharkawi F.Z., Saleh S.S., Sayed A.F.M. (2015). Potential anticancer activity of snake venom, bee venom and their components in liver and breast carcinoma. Int. J. Pharm. Sci. Res..

[21] Cho H.J., Jeong Y.J., Park K.K., Park Y.Y., Chung I.K., Lee K.G., Yeo J.H., Han S.M., Bae Y.S., Chang Y.C. (2010). Bee venom suppresses PMA-mediated MMP-9 gene activation via JNK/p38 and NF-kappaB-dependent mechanisms. J. Ethnopharmacol..

[22] Duffy C., Sorolla A., Wang E., Golden E., Woodward E., Davern K., Ho D., Johnstone E., Pfleger K., Redfern A. (2020). Honeybee venom and melittin suppress growth factor receptor activation in HER2-enriched and triple-negative breast cancer. NPJ Precis. Oncol..

[23] Jung G.B., Huh J.E., Lee H.J., Kim D., Lee G.J., Park H.K., Lee J.D. (2018). Anti-cancer effect of bee venom on human MDA-MB-231 breast cancer cells using Raman spectroscopy. Biomed. Opt. Express.

[24] Yeo S.W., Seo J.C., Choi Y.H., Jang K.J. (2003). Induction of the growth inhibition and apoptosis by beevenom in human breast carcinoma MCF-7 Cells. J. Korean Acupunct. Mox. Med. Sci..

[25] Hematyar M., Soleimani M., Es-Haghi A., Rezaei Mokarram A. (2018). Synergistic co-delivery of doxorubicin and melittin using functionalized magnetic nanoparticles for cancer treatment: Loading and in vitro release study by LC-MS/MS. Artif. Cells Nanomed. Biotechnol..

[26] Moghaddam F.D., Akbarzadeh I., Marzbankia E., Farid M., Khaledi L., Reihani A.H., Javidfar M., Mortazavi P. (2021). Delivery of melittin-loaded niosomes for breast cancer treatment: An in vitro and in vivo evaluation of anti-cancer effect. Cancer Nanotechnol..

[27] LeBeau A.M., Brennen W.N., Aggarwal S., Denmeade S.R. (2009). Targeting the cancer stroma with a fibroblast activation protein-activated promelittin protoxin. Mol. Cancer Ther..

[28] Shaw P., Kumar N., Hammerschmid D., Privat-Maldonado A., Dewilde S., Bogaerts A. (2019). Synergistic effects of melittin and plasma treatment: A promising approach for cancer therapy. Cancers.

[29] Putz T., Ramoner R., Gander H., Rahm A., Bartsch G., Thurnher M. (2006). Antitumor action and immune activation through cooperation of bee venom secretory phospholipase A2 and phosphatidylinositol-(3,4)-bisphosphate. Cancer Immunol. Immunother..

[30] Kanaani L., Javadi I., Ebrahimifar M., Shahmabadi H.E., Khiyavi A.A., Mehrdiba T. (2017). Effects of cisplatin-loaded niosomal nanoparticles on BT-20 human breast carcinoma cells. Asian Pac. J. Cancer Prev..

[31] Kumar G.P., Rajeshwarrao P. (2011). Nonionic surfactant vesicular systems for effective drug delivery—An overview. Acta Pharm. Sin. B.

[32] Edmondson R., Broglie J.J., Adcock A.F., Yang L. (2014). Three-dimensional cell culture systems and their applications in drug discovery and cell-based biosensors. Assay Drug Dev. Technol..

[33] Chitcholtan K., Sykes P.H., Evans J.J. (2012). The resistance of intracellular mediators to doxorubicin and cisplatin are distinct in 3D and 2D endometrial cancer. J. Transl. Med..

[34] Nath S., Devi G.R. (2016). Three-dimensional culture systems in cancer research: Focus on tumor spheroid model. Pharmacol. Ther..

[35] Evans V.G. (1993). Multiple pathways to apoptosis. Cell. Biol. Int..

[36] Shi L., Nishioka W.K., Th’ng J., Bradbury E.M., Litchfield D.W., Greenberg A.H. (1994). Premature p34cdc2 activation required for apoptosis. Science.

[37] Ghilardi C., Chiorino G., Dossi R., Nagy Z., Giavazzi R., Bani M. (2008). Identification of novel vascular markers through gene expression profiling of tumor-derived endothelium. BMC Genom..

[38] Xia Q., Zhang F.F., Geng F., Liu C.L., Xu P., Lu Z.Z., Yu B., Wu H., Wu J.X., Zhang H.H. (2016). Anti-tumor effects of DNA vaccine targeting human fibroblast activation protein α by producing specific immune responses and altering tumor microenvironment in the 4T1 murine breast cancer model. Cancer Immunol. Immunother..

[39] Rahman K.M., Sarkar F.H., Banerjee S., Wang Z., Liao D.J., Hong X., Sarkar N.H. (2006). Therapeutic intervention of experimental breast cancer bone metastasis by indole-3-carbinol in SCID-human mouse model. Mol. Cancer Ther..

[40] Mondal S., Adhikari N., Banerjee S., Amin S.A., Jha T. (2020). Matrix metalloproteinase-9 (MMP-9) and its inhibitors in cancer: A minireview. Eur. J. Med. Chem..

[41] de Melo Gagliato D., Jardim D.L., Marchesi M.S., Hortobagyi G.N. (2016). Mechanisms of resistance and sensitivity to anti-HER2 therapies in HER2+ breast cancer. Oncotarget.

[42] Shah S.P., Roth A., Goya R., Oloumi A., Ha G., Zhao Y., Turashvili G., Ding J., Tse K., Haffari G. (2012). The clonal and mutational evolution spectrum of primary triple-negative breast cancers. Nature.

[43] Den Brok M.H., Nierkens S., Figdor C.G., Ruers T.J., Adema G.J. (2005). Dendritic cells: Tools and targets for antitumor vaccination. Expert Rev. Vaccines.

[44] Perrin-Cocon L., Agaugué S., Coutant F., Masurel A., Bezzine S., Lambeau G., André P., Lotteau V. (2004). Secretory phospholipase A2 induces dendritic cell maturation. Eur. J. Immunol..

